# 518 A Review of the Metabolic Consequences of Illicit Simulant Use in Burn Patients

**DOI:** 10.1093/jbcr/irae036.153

**Published:** 2024-04-17

**Authors:** Louis Ferrari, Billy Jay Taylor, Thomas J Krueger, Jr., Dylon Buchanan, Curt C Bay, Suzanne C Osborn, Claudia Islas, Erica Whetten, Nisha Talanki, Karen J Richey, Kevin N Foster

**Affiliations:** Arizona Burn Center Valleywise Health, Phoenix, AZ; Valleywise Health, Phoenix, AZ; Creighton University, Phoenix, AZ; A.T. Still University, Mesa, AZ; Arizona Burn Center/Valleywise Health Medical Center, Phoenix, AZ; Arizona Burn Center, Phoenix, AZ; Arizona Burn Center Valleywise Health, Phoenix, AZ; Valleywise Health, Phoenix, AZ; Creighton University, Phoenix, AZ; A.T. Still University, Mesa, AZ; Arizona Burn Center/Valleywise Health Medical Center, Phoenix, AZ; Arizona Burn Center, Phoenix, AZ; Arizona Burn Center Valleywise Health, Phoenix, AZ; Valleywise Health, Phoenix, AZ; Creighton University, Phoenix, AZ; A.T. Still University, Mesa, AZ; Arizona Burn Center/Valleywise Health Medical Center, Phoenix, AZ; Arizona Burn Center, Phoenix, AZ; Arizona Burn Center Valleywise Health, Phoenix, AZ; Valleywise Health, Phoenix, AZ; Creighton University, Phoenix, AZ; A.T. Still University, Mesa, AZ; Arizona Burn Center/Valleywise Health Medical Center, Phoenix, AZ; Arizona Burn Center, Phoenix, AZ; Arizona Burn Center Valleywise Health, Phoenix, AZ; Valleywise Health, Phoenix, AZ; Creighton University, Phoenix, AZ; A.T. Still University, Mesa, AZ; Arizona Burn Center/Valleywise Health Medical Center, Phoenix, AZ; Arizona Burn Center, Phoenix, AZ; Arizona Burn Center Valleywise Health, Phoenix, AZ; Valleywise Health, Phoenix, AZ; Creighton University, Phoenix, AZ; A.T. Still University, Mesa, AZ; Arizona Burn Center/Valleywise Health Medical Center, Phoenix, AZ; Arizona Burn Center, Phoenix, AZ; Arizona Burn Center Valleywise Health, Phoenix, AZ; Valleywise Health, Phoenix, AZ; Creighton University, Phoenix, AZ; A.T. Still University, Mesa, AZ; Arizona Burn Center/Valleywise Health Medical Center, Phoenix, AZ; Arizona Burn Center, Phoenix, AZ; Arizona Burn Center Valleywise Health, Phoenix, AZ; Valleywise Health, Phoenix, AZ; Creighton University, Phoenix, AZ; A.T. Still University, Mesa, AZ; Arizona Burn Center/Valleywise Health Medical Center, Phoenix, AZ; Arizona Burn Center, Phoenix, AZ; Arizona Burn Center Valleywise Health, Phoenix, AZ; Valleywise Health, Phoenix, AZ; Creighton University, Phoenix, AZ; A.T. Still University, Mesa, AZ; Arizona Burn Center/Valleywise Health Medical Center, Phoenix, AZ; Arizona Burn Center, Phoenix, AZ; Arizona Burn Center Valleywise Health, Phoenix, AZ; Valleywise Health, Phoenix, AZ; Creighton University, Phoenix, AZ; A.T. Still University, Mesa, AZ; Arizona Burn Center/Valleywise Health Medical Center, Phoenix, AZ; Arizona Burn Center, Phoenix, AZ; Arizona Burn Center Valleywise Health, Phoenix, AZ; Valleywise Health, Phoenix, AZ; Creighton University, Phoenix, AZ; A.T. Still University, Mesa, AZ; Arizona Burn Center/Valleywise Health Medical Center, Phoenix, AZ; Arizona Burn Center, Phoenix, AZ

## Abstract

**Introduction:**

Over the past several decades illicit stimulant (STIM) use has developed into a major, national crisis. At our urban, safety net hospital, this is evident in a consistent increase in patients with chemical evidence of methamphetamine, amphetamine and cocaine use. Adverse health consequences of STIM use are well known, however potential metabolic consequences in patients with significant burns has not been studied. Our hypothesis was that this patient population is both undernourished and hypermetabolic when compared to those who test negative for stimulants.

**Methods:**

This was a retrospective chart review of burn patients over a 6-year period who suffered a burn injury ≥ 20% TBSA and had both toxicology and indirect calorimetry testing completed. Patients were stratified into 2 groups according to toxicology results, positive for stimulants and negative for stimulants. Descriptive statistics were calculated for demographic and outcome data.

**Results:**

A total of 332 patients were admitted over the 6-year period with burn injuries of ≥ 20% TBSA. Among those 140 had both toxicology and indirect calorimetry testing completed. When stratified by toxicology results 34 were positive (POS) and 106 were negative (NEG). There was no significant difference between groups for age 43.21 years POS vs 47.26 years NEG, % TBSA 34% POS vs 35% NEG, or BMI 27.5 kg POS vs 29 kg NEG. Likewise, there were no significant differences in RQ, 0.94 POS vs 0.93, REE 2513 POS vs 2412 NEG or COVAR 5.87 POS vs 5.54 NEG. There was no significant difference in mortality between the two groups 12% POS vs 23% NEG (p=.220).

**Conclusions:**

We were unable to reject the null hypothesis in this study. Nearly two thirds of patients admitted during the study period did not have toxicology testing done and this may have prospectively introduced a selection bias into our retrospective study. Uniformity and protocolization of toxicology testing are necessary to avoid selection bias in order to meaningfully study the potential effects of illicit drugs on the outcomes of burn patients.

**Applicability of Research to Practice:**

While we were unable to demonstrate a significant impact of stimulant use on patient metabolism and nutritional status with this study, it remains our hypothesis that such a relationship exists.

